# Comparison of microstructural and mechanical properties of trabeculae in femoral head from osteoporosis patients with and without cartilage lesions: a case–control study

**DOI:** 10.1186/s12891-015-0530-5

**Published:** 2015-03-31

**Authors:** Houchen Lv, Licheng Zhang, Fei Yang, Zhe Zhao, Qi Yao, Lihai Zhang, Peifu Tang

**Affiliations:** Department of Orthopedics, General Hospital of Chinese PLA, No.28 Fuxing Road, Beijing, China; BNLMS State Key Laboratory of Polymer Physics & Chemistry, Institute of Chemistry, Chinese Academy of Sciences, Beijing, China; Department of Orthopedics, Beijing Shijitan Hospital, Beijing, China

**Keywords:** Osteoporosis, Osteoarthritis, Mechanical properties, Microstructure

## Abstract

**Background:**

Degeneration of cartilage will change load distribution, affecting bone remodeling progress and trabecular structure and strength. However, in human primary osteoporosis, whether cartilage lesions would also affect properties beneath trabecular bone remains unknown. In this study, we explored the differences in local trabecular properties between osteoporosis patients with and without cartilage lesions.

**Methods:**

Eighteen pairs of femoral heads with and without cartilage lesions in a weight-bearing area were collected from senile femoral neck fracture patients. The Mankin score and glycosaminoglycan (GAG) content were used to evaluate the severity of the cartilage lesions. Micro-CT and compression tests were used to obtain structural and mechanical characteristics of each trabecular column. Multivariate linear regression was performed to evaluate the association between mechanical parameters and the degree of cartilage lesion.

**Results:**

In osteoporosis patients with cartilage lesions, the bone volume fraction (BV/TV) and trabecular thickness (Tb.Th) of the trabecular column were significantly higher than that of osteoporotic control patients (all P < 0.05), while the Young’s modulus was lower (P = 0.024). Multivariable linear regression indicated that in both groups, bone mineral density (BMD) significantly correlated with Young’s modulus (all P < 0.05). While in patients with cartilage lesion, GAG content was also correlated with Young’s modulus (standardized coefficient 0.443, P < 0.01).

**Conclusions:**

Osteoporosis patients with cartilage lesions exhibited a weaker mechanical property of trabeculae. The intimate association of cartilage lesions and impairment of trabecular mechanical properties indicate that cartilage and trabeculae belong to an interdependent functional unit. Previously proposed adaptive mechanisms in osteoarthritis might also be applicable to the progression of osteoporosis.

## Background

The relationship between the two age-related skeletal disorders: osteoporosis (OP), characterized by loss of bone mineral and deterioration of microarchitecture [1], and osteoarthritis (OA), characterized by progressive degeneration of articular cartilage and concomitant changes of surrounding bone [2], remains controversial despite decades of study. Previous cross-sectional studies found that the two diseases do not normally occur together in observed populations [3] and some studies suggest an inverse association between OA and OP [4,5].

Indeed, advanced OA (Grade ≥3 or 4) defined by Kellgren-Lawrence (K-L) grade is rarely found in patients with OP (about 2.7% to 4%) [6,7]. However, in these studies, patients diagnosed with OA or advanced OA with severe cartilage lesions can be evaluated by X-ray, while patients with mild cartilage lesions or early stages of OA were not evaluated in this category. Consequently, other research, taking the early stages of OA into account, found that OA and OP could coexist in one person. For example, Healey et al. reported the prevalence of mild OA (Grade ≤2) in osteoporotic women was unusually high, approximately 36% [6]. Moreover, in primary OA patients, the overall rate of osteoporosis (T score < −2.5) and osteopenia (−1.0 > T score > −2.5) was 23-28% and 43-45% respectively in elderly OA patients awaiting arthroplasty [8,9]. While in shoulder osteoarthritis, the incidence of osteoporosis and osteopenia was 12.2% and 42.6% respectively [10]. This research indicates that with further epidemiological validation of the exact proportion of patients with both OA and OP, it may be found that cartilage degradation and osteoporotic changes co-exist within patients, and a comparatively high fraction of these patients might exhibit only mild cartilage lesions [6,8-10].

As cartilage and cancellous bone are the two main components of a human’s weight-bearing system, degradation of cartilage will obviously change the load distribution between cartilage and cancellous bone. Richard et al. reported that the values of viscoelastic parameters were reduced significantly in OA cartilage [11], leading to an impaired energy dissipation capability of cartilage and increasing the incidence of micro-damage beneath cancellous bone. Meanwhile, Chiba et al. found that OA progression accessed by K-L grade even had a correlation with osteoporotic changes in subchondral trabecular bone [12]. In addition, Musumeci et al. found that in a glucocorticoid-induced osteoporosis mice model, an accelerated apoptosis of cartilage cells occurred at the same time [13]. On the other hand, Bellido et al. found less cartilage lesions accompanied by improved structural properties of trabeculae in an OP model after pharmaceutical therapy [14]. The above recent studies all suggest an intimate association of OA progression with trabecular pathological changes.

However, it remains to be determined whether cartilage lesions would also affect the structural and mechanical properties beneath trabecular bone in human primary osteoporosis. In this study, we explored the differences in structural and mechanical properties of local trabeculae between OP patients with and without cartilage lesions, and suggest some correlations between cartilage lesions and trabecular degradation. To minimize the interference of anisotropy of cancellous bone, a self-designed sampling method combining X-ray tomography (CT) with three-dimensional printing (3DP) was used to assure the precise location of a trephine biopsy of trabecular column. We found that the mechanical properties of trabeculae in osteoporosis patients with cartilage lesions were poorer than that of osteoporotic controls, indicating a negative effect of cartilage degeneration on trabecular bone structural and mechanical properties. Identifying the differences of local trabeculae in OP patients with cartilage lesions will not only further the understanding of adaptive mechanism in the progression of arthro-cartilage disease [15], but also give some clinical implications for better prediction of trabecular bone mechanical performance.

## Methods

### Subjects and group setting

Between October 2012 and September 2013, patients with femoral neck fracture caused by mild violence and who had undergone hemiarthroplasty or total arthroplasty were enrolled in this study. Inclusion criteria for the patients were as follows: (1) female, age >50 years, (2) osteoporotic femoral neck fracture, (3) undergone hemiarthroplasty or total arthroplasty, (4) femoral cartilage lesions (Outerbridge classification: stage II or stage III) at the primary weight-bearing area (WBA) [16]. Modified Outerbridge classification, which is traditionally calculated by arthroscopic examination, was used in our study. Details of the classification were as follows: Grade 0 - normal; Grade I - cartilage with softening and swelling; Grade II - a partial thickness defect with fissures on the surface that do not reach subchondral bone or exceed 1.5 cm in diameter; Grade III - fissuring to the level of subchondral bone in an area with a diameter more than 1.5 cm; Grade IV - exposed subchondral bone [17]. Patients with tumors, joint infections, diabetes and other diseases that could impair bone metabolism were excluded. In total, 18 pairs of trabecular columns of the primary stress trabeculae were collected and assigned to two groups. Group 1 consisted of osteoporotic femoral neck fracture without cartilage lesion and Group 2 consisted of osteoporotic femoral neck fracture with OA-like cartilage lesions. Eighteen patients (76.1 ± 9.4 years) with OA-like cartilage lesions were recruited as well as another eighteen age-matched osteoporosis patients (74.2 ± 11.7 years) without cartilage lesions. Bone densities of the contralateral hip were measured by dual energy X-ray absorptiometry (DXA, HOLOGIC Discovery-A, Bedford, MA, USA). The severity of cartilaginous lesions was assessed by Mankin score and GAG content [18]. OP was diagnosed by WHO criteria [1,19]: DXA BMD T-score less than or equal to −2.5 SD, or the presence of 1 or more fragility fractures. Serum levels of P1NP (ng/ml) and β-CTX (ng/ml) were determined by electrochemiluminescence (Cobas E601, Roche Diagnostics, Basel, Switzerland) in the biochemistry department of our hospital. The intra-assay and inter-assay coefficients of variation were 2.8% and 1.6% respectively for P1NP, and 1.8% and 3.2% respectively for β-CTX. All patients signed informed consent agreeing to donate their bone samples and this investigation was approved by the medical ethics committee of the General Hospital of the People’s Liberation Army.

### Specimen preparation

**Figure 1:**
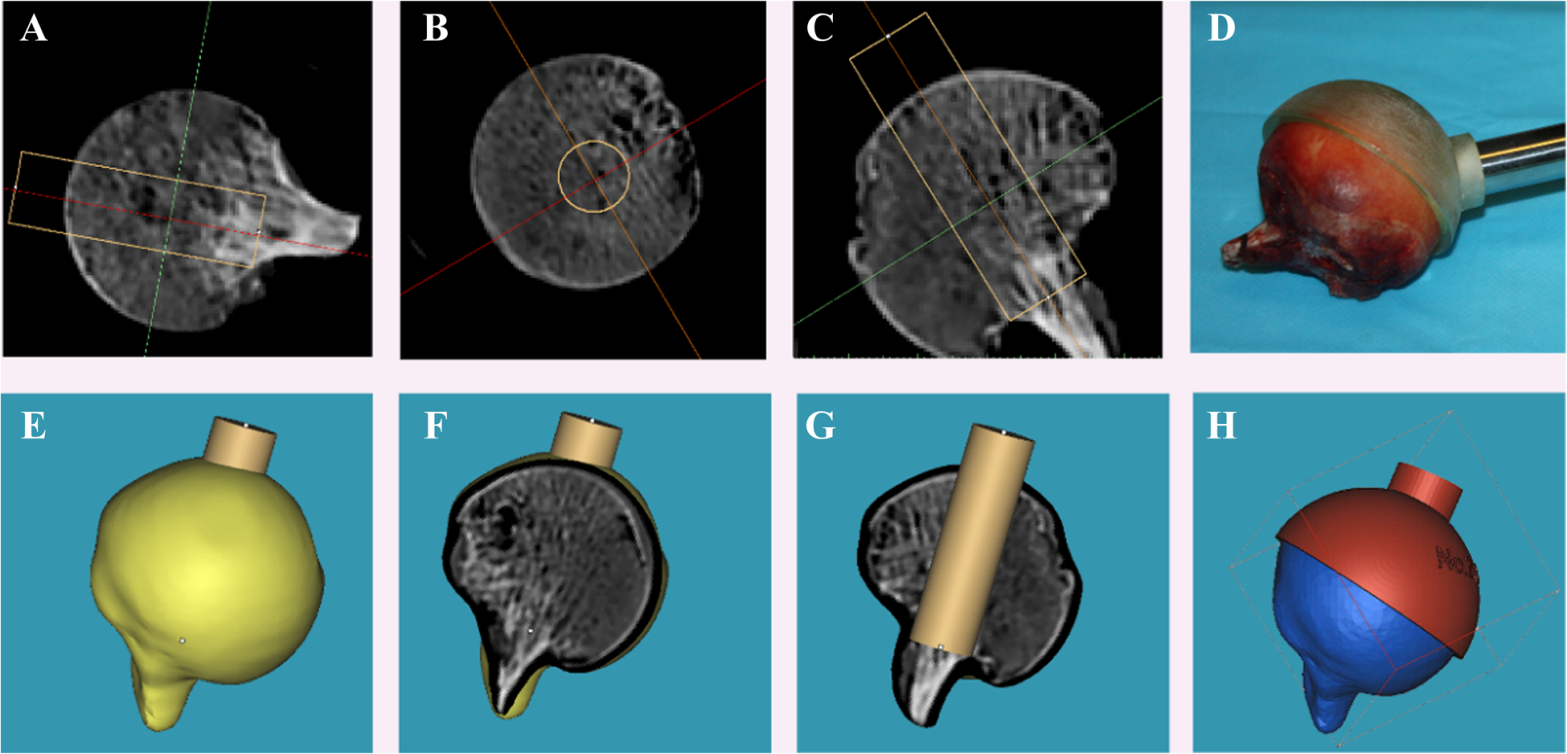
**Steps for manufacture of individualized sampling mold. A**, **B**, and **C** pictures show that primary stress trabeculae is positioned in three different vertical plane. **E**, **F**, and **G** pictures show different section view of the virtual bar represent the primary stress trabeculae. Picture **H** is the schematic of a well-built sampling mold. **D** picture is the real photo captured during sampling operation.

**Figure 2:**
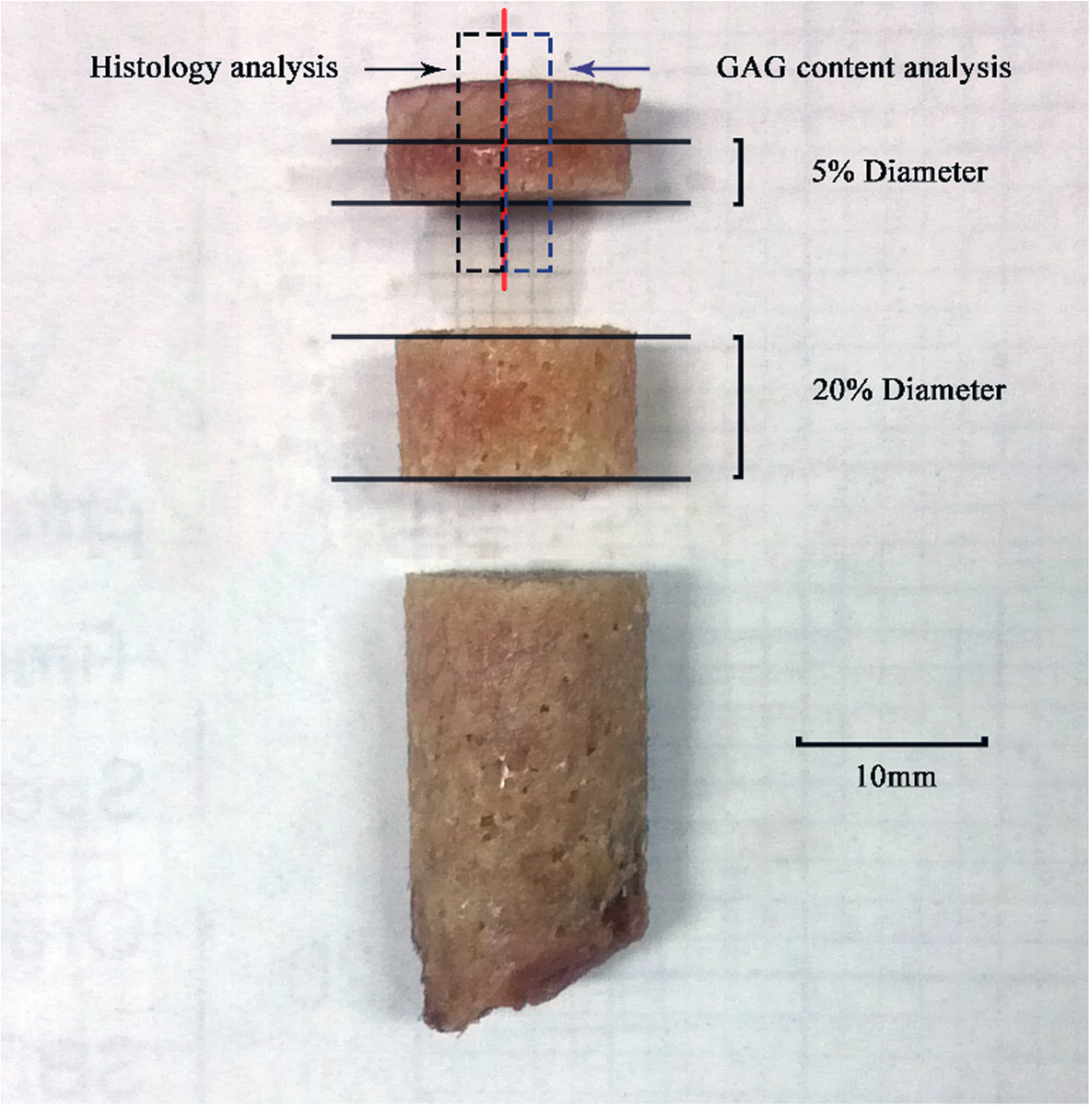
**Illustration of trabeculae column and segmentation of the three parts.** Each column was cut into three segments, using a low speed saw with a diamond cut-off wheel. The first part was used for the evaluation of water content, glycosaminoglycan (GAG) content and histology analysis, about 5% diameter of femoral head. The second part, which was first scanned by Micro-CT and then compressed to obtain mechanical properties which was 20% length of femoral head diameter.

A self-designed sampling method combining X-ray tomography (CT) with three-dimensional printing (3DP) was used to assure the precise location and trephine biopsy of primary compressive trabecular (PCT) columns. Based on the CT scan data, the PCT column was confirmed from three different planes: coronal, sagittal and horizontal planes, as shown in Figure 1 (3-matic 6.0, Materialise, Belgium). A cylinder representing the sampling location was implanted in the primary stress trabeculae arc. Femoral head concave and surface contour were used as reference to create a bowl-shaped mold with a 12 mm diameter needle channel. The designed mold was exported to a 3D printer (OBJET EDEN 260 V, Stratasys Ltd, Rehovot, Israel) in stl format and printed in Transparent Fullcure®720. A personalized mold was made for each femoral head. Trabecular columns with articular cartilage and subchondral bone were prepared using a 12 mm diameter sterile trephine, and stored dry at −80°C. Each column was cut into three segments, using a low speed saw (TechCut 4, Allied Hi-Tech Inc., USA). The length of the first part was 5% of the diameter of the femoral head; the second part was 20% percent (Figure 2). As this study was not designed to explore the regional differences in trabecular columns or depth dependence as described previously [20], we chose columns for the mechanical test at the relative position of the primary compression arc, at the second part (with a height of 20% of the head diameter adjusted by head diameter) [21,22]. The first segment was used for cartilage evaluation, including water content, GAG content, and histology analysis; the second segment was first scanned by Micro-CT and then compressed to obtain mechanical data.

### Micro-CT and mechanical tests of trabecular columns

The trabecular column of each patient was scanned on micro-CT (SkyScan1076 in-vivo micro-CT, Bruker microCT, Kontich, Belgium) using parameters defined by Sun [23]. All scans were in the mode of 70 kV X-ray voltage, 140 μA current, 1 mm aluminum filter, and a rotation step of 0.6°_,_ 180° rotation. A calibration scan was taken with two CaHA rods of 0.25 and 0.75 g/cm^−3^ in a water tube prior to each measurement. All scans were reconstructed with the same parameters using Skyscan Recon software (version 1.6.4.1, Bruker microCT). Three-dimensional structural parameters including apparent BMD (HA g/cm^3^), bone volume fraction (BV/TV), trabecular thickness (Tb.Th), trabecular number (Tb.N), trabecular separation (Tb.Sp), structure model index (SMI), degree of anisotropy (DA), and Connectivity density (Conn.D) of the region of interest (ROI) were calculated using CTan software (version 1.11.8.0, Bruker microCT).

### Mechanical tests of trabecular columns

After the micro-CT scan, vertical unconfined compression tests were performed for each trabecular column. Each column was compressed in the inferosuperior direction between two platens at the speed of 1%/min on Instron 3366 10-kN Dual Column Testing Systems (Instron, High Massachusetts, USA). A stress–strain curve was plotted as the test progressed and three parameters, including Young’s modulus, yield strength and ultimate strength were calculated to describe the mechanical properties of the trabecular columns.

### GAG analysis

A dimethylmethylene blue spectrophotometric assay developed by Farndale et al. [24] was used to determine the levels of GAG in papain-digested cartilage specimens [25-27]. The first part of the trabecular column was further divided into two equal parts; cartilage randomly prepared from one of the two parts was used to measure water content by lyophilization. After recording the dry weight, the cartilage sample was immersed in a 20 ml papain enzyme solution which consisted of 35 mg L-cysteine (Energy Chemical, A070052, Shanghai, China) in 20 ml phosphate-buffered ethylene diamine tetraacetic acid (PBE) and 0.1 ml papain (Sigma Aldrich, 76218, St. Louis, MO, USA) at an activity of 35 U/mg. 3 ml of this solution was added to the cartilage sample in a 5 ml round-bottom flask, sealed with polyethylene film, and placed in an oil bath at 60°C-65°C for about 16 hours until the cartilage sample was completely dissolved. Standard cure was made by diluting chondroitin-sulphate-4 (Sigma Aldrich, C4384, St. Louis, MO, USA) using PBE-cysteine solution to reach concentrations of 2.5, 5, 10, 25, 50 and 100 μg/ml. Color reagent was prepared by dissolving 16 mg dimethylmethylene blue (Sigma Aldrich, 341088, St. Louis, MO, USA) in 1 L water containing 3.04 g glycine, 2.37 g NaCl and 95 ml of 0.1 mol/L HCl at pH 3.0. 100ul of standard chondroitin sulphate solution or cartilage sample solution was added to 2.5 ml dimethylmethylene blue color reagent in a 3 ml cuvette. Spectrophotometric measurements were taken immediately at 520 nm in triplicate. The concentration of the sample was calculated automatically (TU-1901, Ultraviolet–visible spectrophotometry, Persee, China). Values of GAG content are expressed as micrograms per milligram dry weight of cartilage tissue in WBA.

### Histopathological analysis

Another part of the trabecular column with associated cartilage sample was fixed in 10% formaldehyde solution for 48 hours and decalcified with 10% ethylene diamine tetraacetic acid (EDTA) for 4 weeks according to standard protocols. Cartilage slices were stained with Safranin O/Fast Green. Three histologic slices of each sample were graded by two experienced pathologists according to Mankin’s grading system [17,18,28].

### Statistical analysis

Results were expressed as the mean and standard deviation of the mean. Normality of the distribution was assessed by Kolmogorov-Smirnov test. For variables that did not have a normal distribution, the median with 25%-75% interquartile range was used. Differences between the 2 groups were analyzed using Student’s t-test or Mann–Whitney U test. To determine the potential association between mechanical and other parameters, Young’s modulus, PINP, β-CTX, BMD, BV/TV, Tb.Th, Tb.N, Tb.Sp, SMI index and Conn.D were analyzed by bivariate correlations to detect a linear association in each group. Pearson correlation coefficients (r) and significance levels (*P*) were calculated and multiple linear regression analysis was used to evaluate which parameter was most significantly associated with mechanical properties. A stepwise method was used to calculate the value of r^2^. All statistical analyses were performed using SPSS 19.0 software (IBM Corporation, Armonk, NY) and a P value of <0.05 was considered significant.

## Results

### Baseline characteristics

Baseline clinical and demographic characteristics of the two groups are shown in Table 1. No significant differences were found in menopausal age, body weight, body height, BMI, diameter of femoral head, BMD of contralateral femoral neck or β-CTX between the two groups (P > 0.05). However, the Mankin score and PINP were significantly different between the two groups.

**Table 1:** **Baseline clinical and demographic characteristics of two groups**

**Variable**	**Group 1 (N = 18)**	**Group 2 (N = 18)**	***P*** **value**
Age (years)	74.2 ± 11.7	76.1 ± 9.4	0.164
Menopausal age	49.9 ± 2.3	50.1 ± 2.8	0.669
Body weight (kg)	67.6 ± 16.0	61.8 ± 16.3	0.267
Body height (cm)	167.2 ± 8.5	164.3 ± 10.6	0.419
BMI	24.0 ± 4.7	22.8 ± 5.5	0.461
Thickness in WBA (mm)	1.84 ± 0.67	1.76 ± 0.52	0.684
Diameter of femoral head (cm)	5.13 ± 0.44	4.92 ± 0.43	0.153
Mankin score	2(1,6)	10(9,11)	0.000*^a^
BMD (femoral neck) (g/mm^2^)	0.628 ± 0.10	0.680 ± 0.09	0.169
T-SD	−2.1 ± 0.8	−1.7 ± 0.7	0.176
PINP (ng/ml)	38.1 ± 11.1	54.7 ± 18.7	0.003*
β-CTX (ng/ml)	0.55 ± 0.15	0.64 ± 0.35	0.324

Group 1: OP control group, Group 2: OP with Cartilage lesions. Statistically significant difference at the level *P* < 0.05*, P value column was calculated by student’s t test. ^a^Mankin score were analyzed by Mann Whitney U test, data was expressed as median with 25%-75% interquartile range.

### Evaluation of cartilage lesions

**Figure 3:**
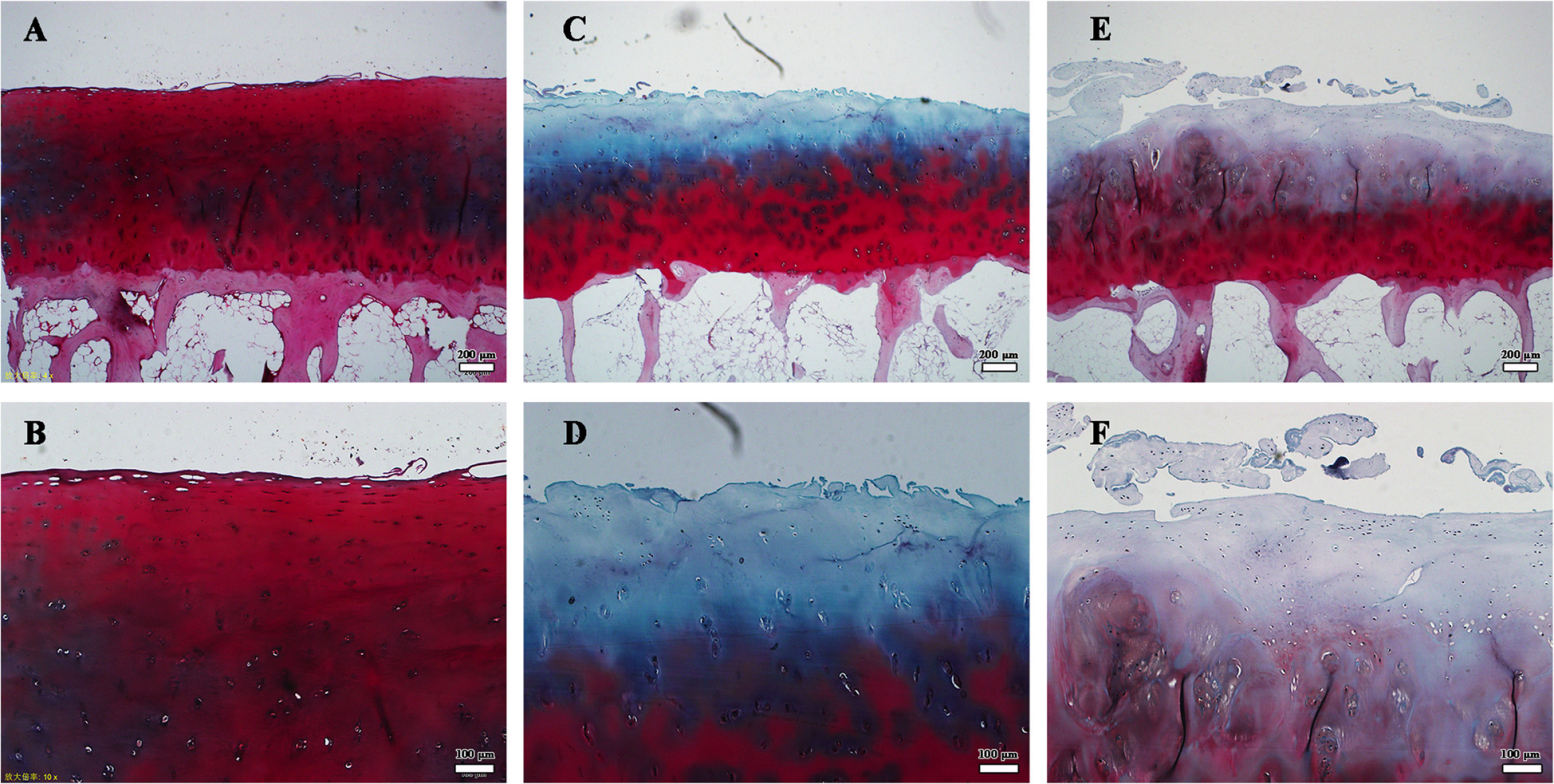
**Histopathological evaluation of Safranin O/Fast Green stain. A**, **B**. Stains from patient of Outerbridge 1 level cartilage lesion, cartilage of WBA in the same patients shows homogeneous and intense staining of proteoglycans at extracellular matrix, normal structure and normal cellularity across the different layers. **C**, **D**. Outerbridge 2 level, cartilage shows reduced safranin O staining of extracellular matrix, cells in upper layer shows hypercellularity and cartilage surface irregular. **E**, **F**. Outerbridge 3 level, cartilage displays severely reduced safranin O staining of extracellular matrix, cell cloning cellularity and hypocellularity, and clefts to transitional zone.

The severity of cartilage lesions in the two groups were compared using three different methods, as shown in Table 2. In Group 2, the cartilage lesions were more severe than those in Group 1, as measured by Outerbridge classification, Mankin score or GAG content. In Group 1, GAG content and water content were higher than in Group 2 (GAG content:18.3 ± 5.1% vs 15.1 ± 3.8%, P < 0.05, water content: 56.9 ± 9.7% vs 49.1 ± 10.1%, P < 0.05). Safranin O/Fast Green stain of Outerbridge 1, 2 and 3 are shown in Figure 3. In Group 1, the Mankin score was significantly lower than that in Group 2 (P <0.001). Details of the stain result are shown in Table 2.

**Table 2:** **Comparison of severity of cartilage lesions in the two groups by Mankin score, Modified Outerbridge classification and GAG content**

**Variable**	**Mankin score**			**Outerbridge classification grade**				**GAG content (%)**	**Water content (%)**
	**<6**	**6-10**	**>10**	**1**	**2**	**3**	**4**		
Group 1	12(1–2)	6(6–8)	0	18	0	0	0	18.3 ± 5.1	56.9 ± 9.7
(N = 18)									
Group 2	0	10(6–10)	8(11–12)	0	12	6	0	15.1 ± 3.8	49.1 ± 10.1
(N = 18)									

Group 1: OP control group, Group 2: OP with Cartilage lesions. Reported values in the parentheses are ranges. Significant differences were found in the two groups for GAG content and water content (all P < 0.05).

### Mechanical evaluation of trabeculae

Mechanical and structural parameters are shown in Table 3. Young’s modulus was significantly higher in Group 1 than in Group 2 (248.4 ± 77.3 MPa vs. 192.5 ± 64.4 MPa, P < 0.05). Yield strength and ultimate strength also show significant differences between the two groups. BV/TV in Group 1 was lower than in Group 2 (16.7 ± 4.4% vs. 20.7 ± 6.9%, P < 0.05). Tb.Th in Group 1 was thinner than in Group 2 (0.17 ± 0.03 mm vs. 0.20 ± 0.06 mm, P < 0.05). Other parameters, including BMD, BS/TV, BS/BV, Tb.N, Tb.Sp and Conn.Dn displayed no significant differences between the two groups.

**Table 3:** **Mechanical and structural properties of the two groups**

**Variable**	**Group 1**	**Group 2**	***P value***
	**OP control (N = 18)**	**OP with cartilage lesions (N = 18)**	
**Mechanical properties**			
Young’s modulus (MPa)	248.5 ± 77.3	192.5 ± 64.4	0.024*
Yield strength (MPa)	5.0 ± 2.3	3.6 ± 1.3	0.032*
Ultimate strength (MPa)	6.7 ± 2.8	4.6 ± 1.4	0.005*
**Trabeculae microstructural parameters**			
BMD	0.26 ± 0.10	0.30 ± 0.08	0.207
BV/TV (%)	16.7 ± 4.4	20.7 ± 6.9	0.044*
BS/BV (mm^−1^)	21.8 ± 5.2	19.2 ± 7.0	0.219
BS/TV (mm^−1^)	3.5 ± 0.6	3.6 ± 0.5	0.477
Tb.Th (mm)	0.17 ± 0.03	0.20 ± 0.06	0.041*
Tb.Sp (mm)	0.86 ± 0.14	0.80 ± 0.12	0.141
Tb.N (mm^−1^)	0.99 ± 0.18	1.03 ± 0.14	0.373
SMI	0.72 ± 0.39	0.72 ± 0.44	0.998
DA	2.56 ± 1.10	2.10 ± 0.33	0.101
Conn.Dn (mm^−3^)	3.53 ± 1.58	2.77 ± 1.42	0.139
BMD_Neck_	0.63 ± 0.10	0.68 ± 0.09	0.102

BMD means apparent density, representing the mean density of selected column VOI of the bone column was calculated by CTan and BMD_Neck_ means density of the contralateral femoral neck collected by DEXA. Statistically significant difference at the level *P* < 0.05*.

Young’s modulus had a linear correlation with BMD in both groups (both P < 0.05). In Group 1, Young’s modulus also positively correlated with BV/TV (r = 0.750, P < 0.05) and Th.N (r = 0.688, P < 0.05) and negatively correlated with SMI (r = −0.516, P < 0.05). In Group 2, Young’s modulus was also significantly correlated with BV/TV (r = 0.707, P < 0.05) and Tb.N (r = 0.797, P < 0.05). Meanwhile, Young’s modulus also negatively correlated with Tb.Sp (r = −0.535, P < 0.05), but not with SMI (r = −0.405, P = 0.095). No association was found with PINP and β-CTX in either group. Young’s modulus positively correlated with GAG content in both groups, especially in Group 2 (Details are shown in Table 4). The higher correlation coefficient between Young’s modulus and GAG content in Group 2 indicates that decreased mechanical properties of subchondral trabeculae may be associated with the degradation of cartilage in this particular subgroup population, osteoporosis patients with OA-like cartilage lesions.

**Table 4:** **Correlation analysis of Young’s modulus and structural parameters in Micro-CT**

**Young’s modulus**		**PINP**	**β-CTX**	**GAG content**	**BMD**	**BV/TV**	**Tb.Th**	**Tb.Sp**	**Tb.N**	**SMI**	**Conn.Dn**
Group 1	r	0.048	−0.01	0.473*	0.898**	0.750**	0.421	−0.454	0.688**	−0.516*	−0.019
(N = 18)	P	0.85	0.968	0.047	0.001	0.001	0.082	0.058	0.002	0.028	0.94
Group 2	r	−0.006	−0.315	0.785**	0.877**	0.707**	0.497*	−0.535*	0.797**	−0.405	−0.33
(N = 18)	P	0.982	0.203	0.001	0.001	0.001	0.036	0.022	0.001	0.095	0.181

Group 1: OP control group, Group 2: OP with Cartilage lesions. *P <0.05, **P < 0.001, Nonparametric correlation was used for the analysis between Young’s modulus and SMI.

### Association of GAG and mechanical properties

**Figure 4:**
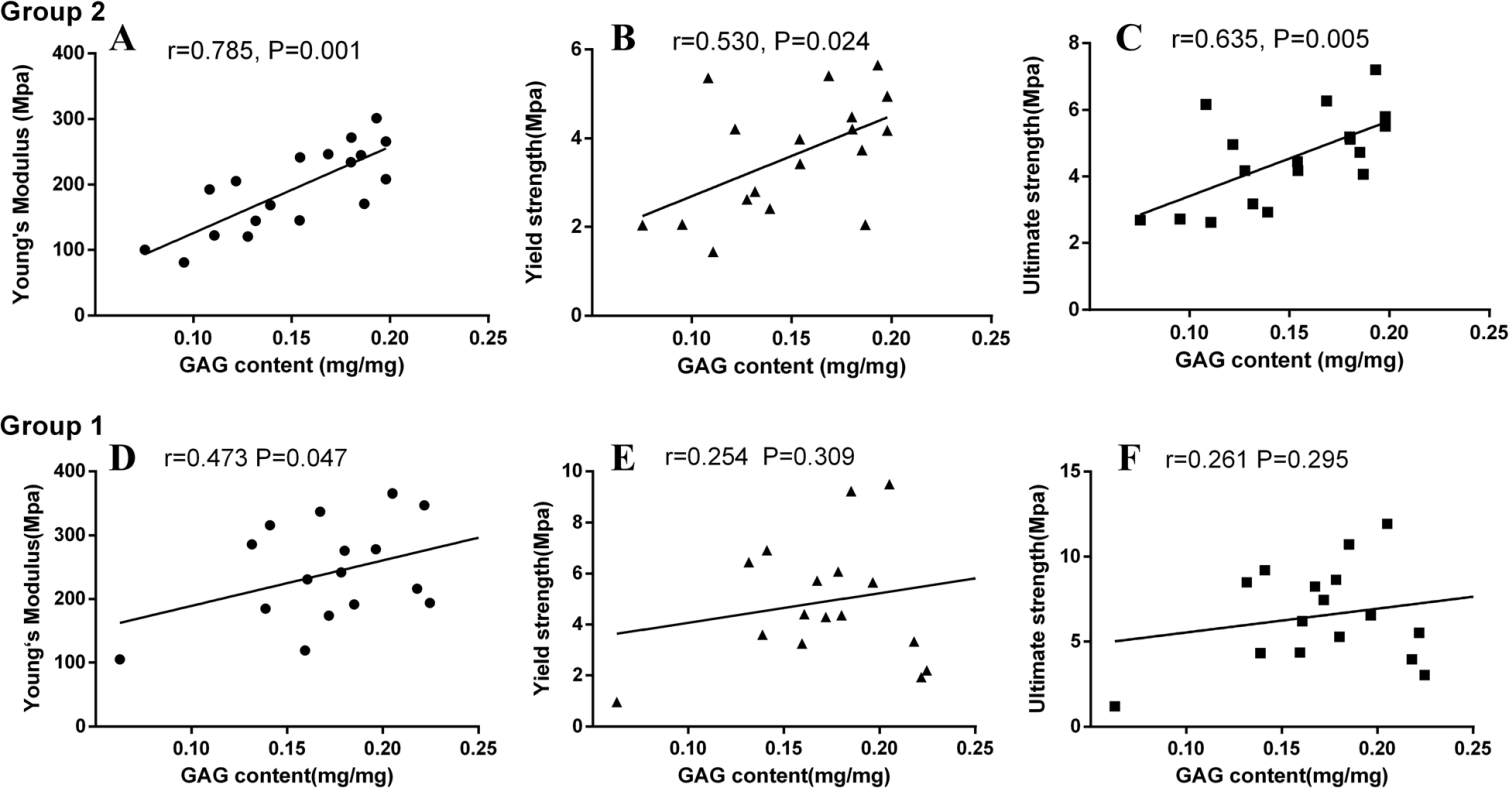
**Correlation between GAG content and Young’s modulus, yield strength and ultimate strength.** In Group 2 (with cartilage lesions), mechanical properties all correlated with GAG content. **A**, Young’s modulus positively correlated with GAG content (r=0.785, P=0.001). **B**, Yield strength positively correlated with GAG content (r=0.530, P=0.024). **C**, Ultimate strength positively correlated with GAG content (r=0.635, P=0.005). In Group 1 (without cartilage lesions), **D**, Young’s modulus positively correlated with GAG content (r=0.473, P=0.047). **E**, Yield strength not significantly correlated with GAG content (r=0.254, P=0.309). **F**, Ultimate strength not significantly correlated with GAG content (r=0.261, P=0.295). These figures indicate that decreased mechanical property of trabeculae may associated with the degradation of cartilage in osteoporosis patients with cartilage lesions.

GAG content was further analyzed with the other mechanical parameters by Pearson’s correlation coefficient within each group. In Group 2, the GAG content exhibited a linear correlation with yield strength (r = 0.530, *P* < 0.05) and ultimate strength (r = 0.635, *P* < 0.05). In Group 1, no significant association was detected (Figure 4). Multivariate linear regression shows that BMD could predict the variance of Young’s modulus [Model: r^2^ = 0.850, P < 0.01; Beta _BMD_ (Standardized coefficient): 0.710, P = 0.003] in Group 1. However, in Group 2, GAG content and BMD could predict the variance of Young’s modulus (Model: r^2^ = 0.917, P < 0.01, Beta _GAG content_: 0.443; Beta _BMD_:0.645, all P < 0.01) (Table 5).

**Table 5:** **Multiple regression models for predicting Young’s modulus for two groups**

Model*		**Unstandardized coefficients**		**Standardized coefficients**	**t**	**P**
		**B**	**Std.Error**	**Beta**		
Group 1	Constant	16.408	101.197		0.16	0.874
	BMD	534.452	142.100	0.710	3.76	0.003*
	GAG content	220.224	193.024	0.146	1.14	0.276
	Age	0.699	0.853	0.106	0.82	0.428
	BV/TV	6.489	4.284	0.366	1.51	0.156
	Tb.Th	−640.501	563.204	−0.257	−1.14	0.278
Group 2	Constant	−65.437	66.019		−0.99	0.341
	BMD	515.321	100.005	0.645	5.15	0.000*
	GAG content	746.284	169.159	0.443	4.41	0.001*
	Age	0.206	0.794	0.030	0.26	0.800
	BV/TV	0.333	2.332	0.036	0.14	0.889
	Tb.Th	−159.956	301.933	−0.138	−0.53	0.606

*significance was set at the P = 0.05 level of probability for inclusion in the model.

## Discussion

In previous studies, researchers mostly concentrated on the comparison of microstructural features of cancellous bone in OP and OA patients and cadaver controls [12,29-32]. Whether cartilage lesions would also affect the structural and mechanical properties of trabecular bone in osteoporosis was unclear. In this study, we adopted a case–control study design to address this issue. The primary result was that the mechanical properties of trabeculae in osteoporosis patients with cartilage lesions were diminished as compared to osteoporotic controls, indicating a negative effect of cartilage degeneration on trabecular bone in osteoporosis patients.

Many previous studies have explored structural and mechanical properties of femoral head trabeculae in different diseases, particularly OA and OP [1,22,32-34]. Most studies chose the trabeculae, which are located at the principal compressive trabecular arc and extend to medial calcar. However, no satisfying solution was given for accurate location of the target cancellous bone column. It is known that structural characteristics of cancellous bone in the femoral head vary from different sites [35,36]. Extraction of the trabecular column in corresponding positions from different individuals is the prerequisite for follow-up experiments. However, femoral heads collected during hemiarthroplasty or total arthroplasty are all separated from the femoral neck, trochanter and lesser trochanter. It is quite difficult to distinguish the anterior, posterior, lateral or medial head only by visual inspection. Additionally, it is difficult to extract a sample column in the same location in a different femoral head. To solve this technical problem, we designed a personalized mold for each femoral head with the help of a CT and 3DP technique to ensure the precise location and trephine biopsy of the target trabeculae column. Steps for the manufacture of the mold are shown in Figure 1. By adjusting the size and passage direction from different cross sections, this method can help draw out any portion of cancellous bone in femoral head. This method can dramatically improve the accuracy and comparability of trabecular columns, and has a great advantage over the conventional visual inspection method.

In our study, BV/TV and Tb.Th of trabecular columns were higher in femoral heads with cartilage lesions (Group 2) than in femoral heads without (Group 1), as shown in Table 3. This result is consistent with previous studies about histomorphometric parameters of OP and OA cancellous bone. Dai et al. had reported that BV/TV in OA patients was higher than that in osteoporosis patients (34.83 ± 11.85% vs. 19.62 ± 2.54%, P < 0.01) [30]. Aspden et al. also reported that there was a 72% higher volume of trabecular bone in OA patients, and a 20% lower volume of trabecular bone in osteoporosis patients compared to normal controls [31]. Meanwhile, Bobinac et al. reported that trabecular bone from a group with a lower total Mankin score had a significantly lower BV/TV value than samples from a group with a higher Mankin score [32]. While in OA patients bone sclerosis is associated with an increase of osteoid substance deposition [37], higher values of BV/TV in osteoporotic patients with OA-like cartilage lesions might be attributed to similar pathological changes of OA. This increased bone formation is supported by the higher PINP in Group 2 in our study. As for the mechanical properties, we found all three mechanical parameters: Young’s modulus, yield strength, and ultimate strength were lower in patients with cartilage lesions than in the other group. Further correlation analysis showed that Young’s modulus exhibited a positive associations with GAG content in both groups, especially in patients with cartilage lesions (r = 0.785, P = 0.001). Previous research suggested that increased osteophytes in advanced OA would result in an increased degree of mineralization, enhancing the mechanical properties of cancellous bone [21]. However, in the present study, we showed that osteoporosis patients with OA-like cartilage lesions have diminished mechanical properties compared with the control group. When structure changes were taken into account, this clearly different mechanical behavior might indicate that osteoporosis patients with OA-like cartilage lesions have different pathological changes during disease progression. In the OP control group, Young’s modulus negatively correlated with SMI (r = −0.516), which was not found in patients in the cartilage lesion group in our study. This difference implies that the increased bone formation or mineral sedimentation may increase the mineral content, but not necessarily lead to regularly arranged hybrid structures of collagen and mineral. Further research about subchondral bone plate degeneration, trabeculae mineralizing pattern, micro-fracture density and metabolic changes of local bone are needed to give a comprehensive explanation about the reduced mechanical properties in osteoporotic femoral neck fracture patients with cartilage lesions.

In addition, multivariable regression analysis indicated that BMD was significantly associated with Young’s modulus in both groups (Table 5). And GAG content was another important factor that was associated with Young’s modulus in patients with cartilage lesions (Standardized determination coefficient GAG content: 0.443) (Table 5). It has already been confirmed that BMD is one of the important factors affecting bone mechanical properties [38]. Our results indicate that in osteoporosis patients with OA-like cartilage lesion, the degree of cartilage lesions might also be used as an indicator to predict mechanical properties beneath trabecular bone. This is of significance in clinical practice: if cartilaginous lesions are detected in one side of the femoral head during prosthetic replacement surgery for OP fracture, there might be an increased likelihood of trabecular microcrack or osteoporotic fracture of contralateral proximal femur. The intimate association of cartilage lesions and impairment of mechanical properties of trabeculae indicate that cartilage and trabeculae belong to an interdependent functional unit. So previously proposed adaptive mechanisms in OA might possibly be extended to this interdependent functional unit [15]. Further research about the interrelationship of the two components are need to validate this hypothesis.

We acknowledge that there remains some limitations in our study. First, the sample size could be further extended. Although we strictly established the selection criteria of participants and used a more accurate sampling method, the sample size was not enough for accurate multiple variable linear regression analysis taking BMD, GAG content and structural parameters as independent variables. Further larger sample size studies taking structural parameters into consideration are need to validate this result. Second, due to defects of early design, we did not include patients of OA without osteoporosis, so here we cannot assess how the differences in severity of OA affect underlying bone in patients with or without osteoporosis. Third, structural and mechanical properties of trabeculae were assessed only in a portion of the principal compressive trabecular arc. Other parts of the femoral head trabeculae should also be studied to obtain a comprehensive evaluation of osteoporotic status of cancellous bone. The last two deficits can be amended in future studies to further confirm our conclusions.

## Conclusions

After comparing the properties of trabeculae in osteoporosis patients with and without cartilage lesions, we conclude that the degree of cartilage degradation in these patients is closely related to its mechanical properties. Osteoporosis patients with cartilage lesions exhibited weaker mechanical properties of trabeculae, thus may have an increased vulnerability to trabecular microcrack or even osteoporotic fracture. During progression of cartilage lesions, impairment of mechanical properties of trabeculae might also occur at the same time, indicating cartilage and trabeculae belong to an interdependent functional unit. Previously proposed adaptive mechanisms in OA might possibly be extended to this interdependent functional unit and to the progression of osteoporosis.

## Abbreviations

OA: Osteoarthritis
OP: Osteoporosis
CT: X-ray tomography
3DP: Three-dimensional printing
GAG: Glycosaminoglycan content
K-L grade: Kellgren-Lawrence grade
PCT: Primary compressive trabeculae
DXA: Dual energy X-ray absorptiometry
BMD: Bone mineral density (calculated by Micro-CT)
BMD_neck_: Bone mineral density (measured by DXA)
BV/TV: Bone volume fraction
Tb.Th: Ttrabecular thickness
Tb.N: Trabecular number
Tb.Sp: Trabecular separation
SMI: Structure model index
DA: Degree of anisotropy
Conn.D: Connectivity density
ROI: Region of interest
